# Feedback-Seeking Behavior and Its Synergistic Association With Emotional Intelligence: A Study Among Undergraduate Students at a College in Chennai, India

**DOI:** 10.7759/cureus.85963

**Published:** 2025-06-13

**Authors:** Susila T, Evangeline Mary A, Seenivasan P, Tamilarasi R, Kanimozhi R, Punitha Kumari

**Affiliations:** 1 Department of Community Medicine, Stanley Government Medical College, Chennai, IND; 2 Department of Community Medicine, Kovai Medical Center and Hospital (KMCH) Institute of Health Sciences and Research, Coimbatore, IND

**Keywords:** emotional intelligence, feedback-seeking behavior, medical students, quick emotional intelligence self-assessment test, self-efficacy

## Abstract

Background

Emotional intelligence (EI) is the capacity of an individual to comprehend and react to their own and others’ emotions, utilizing this understanding to shape their thoughts and actions. There has been a growing interest in the significance of EI in the realm of effective clinical practice.

Objectives

This study aimed to assess EI and feedback-seeking behavior among medical students at a college in Chennai, India, and to identify the demographic factors influencing these constructs.

Methodology

This cross-sectional study was conducted among 159 undergraduate medical students from November 2022 to January 2023. Data was collected using a pretested, structured questionnaire that included sections on socio-demographic details, the Quick Emotional Intelligence Self-Assessment Test, and feedback-seeking behavior. Statistical analysis was performed using IBM SPSS Statistics, version 21.0 (IBM Corp., Armonk, NY).

Results

EI scores ranged from 57 to 160, with a mean of 108.69 ± 18.35. Out of 159 students, 109 (68.6%) had a satisfactory EI score (25-34) and 40 (25.2%) had a good EI score (35-40). Relationship management was significantly better among females compared to males. Day-scholars exhibited significantly better emotional management skills than hostelites. Students with effective relationship management and social-emotional awareness acknowledged that feedback was a key support for learning. Furthermore, stronger relationship management was associated with increased motivation to receive feedback and a stronger desire to seek it.

Conclusions

One in four students scored below 24, indicating a potential area for enrichment in EI, thus demanding attention and development in this area.

## Introduction

Emotional intelligence (EI) is the ability to manage one’s own emotions and understand the emotions of those around them [1]. The key elements of EI include self-awareness, self-regulation, motivation, empathy, and relationship management [2]. The “emic” perspective of EI enables an individual to understand and regulate their own emotions for effective human interactions, while the “etic” perspective allows them to relate to, empathize with, and respond appropriately to the emotions of others [1]. 

In the medical profession, where human interaction is crucial, EI plays a vital role. It enables healthcare professionals to empathize with patients, which not only enhances clinical interactions but also contributes to improved clinical outcomes. By fostering empathy and compassion, healthcare providers help patients perceive a sense of well-being, which can positively impact their recovery [1].

EI also plays a pivotal role in enabling physicians to work effectively as part of a multidisciplinary team comprising nurses, hospital administrators, and allied health professionals [1]. Moreover, EI is essential for coping with the stressful demands of a medical career, enhancing job satisfaction, and improving performance [3]. In today’s healthcare landscape, where patient satisfaction is a key metric of success, a doctor’s EI becomes a vital asset. High EI is positively correlated with academic success, social skills, strong interpersonal relationships, and the ability to manage stress. Conversely, low EI has been linked to deviant behavior, substance abuse, and poor interpersonal relationships [4].

The rising incidence of violence against doctors and health care professionals in recent times is a serious concern. Factors such as the vulnerability of patients and their families, uncertainty surrounding treatment outcomes, overcrowded hospitals, and overburdened healthcare providers contribute to these episodes. A lack of EI among medical professionals can exacerbate these issues, leading to greater frustration among patients and their families, which can escalate into violence [1].

Feedback is a critical component of medical education, as it enhances performance and fosters self-regulated learning. While feedback is primarily provided by educators, the act of receiving and seeking feedback is predominantly student-driven, reflecting their role as active learners [5]. Feedback-seeking behavior is influenced by self-assessment skills, learning motivation, and the teacher-student relationship [6]. A student’s ability to manage emotions significantly influences their receptiveness to feedback, even when it challenges their sense of identity. In health professions education, integrating feedback is essential for improving knowledge, skills, and attitudes, particularly in experiential learning environments such as clinical settings. The capacity to process and internalize feedback is vital for developing professional competency and ensuring patient safety [7,8]. Students with higher EI are more adept at regulating negative emotions from critical feedback, such as disappointment or anxiety, which in turn fosters a greater openness to feedback. Cultivating EI and encouraging feedback-seeking behavior can thus help establish a constructive feedback culture, where students actively seek, process, and use feedback to enhance academic performance [9,10].

EI and feedback-seeking are recognized as essential competencies in medical education, contributing to the development of empathetic and competent physicians. However, the interplay between these two constructs remains insufficiently explored, particularly within the Indian undergraduate context. This study aims to bridge this gap by examining the association between EI and feedback-seeking behavior among medical students and identifying the demographic factors that may influence them.

Objectives 

1. To assess the levels of EI and feedback-seeking behavior among medical students.

2. To examine the socio-demographic factors associated with EI and feedback-seeking behavior.

3. To analyze the relationship between EI and feedback-seeking behavior.

## Materials and methods

This cross-sectional study was conducted at the Government Stanley Medical College and Hospital, Chennai, India, over a period of three months, from November 2022 to January 2023. The study population consisted of third-year undergraduate medical students. All consenting students were included in the study, while those who submitted incompletely filled questionnaires were excluded.

Sampling and sample size

Census sampling was used as the sampling method. The sample size was calculated using the formula: n = Z²SD²/d², where SD = 16.44 [1] and d = absolute precision of 3. This resulted in a sample size of 115. After accounting for a 20% non-response rate, the final required sample size was 138.

Study tools

EI among students was assessed using the “Quick Emotional Intelligence Self-Assessment Test,” which evaluates four key domains: emotional awareness, emotional management, social-emotional awareness, and relationship management. The total EI score ranges from 0 to 160, with each domain contributing a score between 0 and 40. Scores were categorized as follows: 0-24 indicates a poor score (area for enrichment), requiring attention and development; 25-34 denotes a satisfactory score (effective functioning), where further strengthening is recommended; and 35-40 reflects a good score (enhanced skills), suggesting that the individual can leverage this domain to support others [11].

Feedback-seeking behavior was evaluated using a set of six independent questions developed based on the “Health Belief Model.” These questions assessed the following dimensions:

1. Perceived susceptibility: willingness to engage with feedback even if it causes discomfort.

2. Perceived intensity: belief that feedback is as important for learning as lectures or tutorials.

3. Perceived benefits: recognition of feedback as a valuable resource to support learning.

4. Perceived barriers: attitude that minimal or no significant action is needed after receiving feedback.

5. Cues to action: perception of feedback as motivating.

6. Self-efficacy: intention to actively seek feedback.

Prior to conducting the survey, both the Quick Emotional Intelligence Self-Assessment Test and the feedback-seeking behavior scale (developed based on the Health Belief Model) were reviewed by a clinical psychologist and a medical education expert to ensure content validity in the local context. Additionally, the scales were pre-tested among medical undergraduates to assess clarity, relevance, and contextual appropriateness.

Data collection procedure

Permission was obtained from the Institutional Ethics Committee, Government Stanley Medical College and Hospital, Chennai, India (Dt13092019/IEC/SMC). Participants were informed about the study, and written informed consent was obtained. Self-administered printed questionnaires were distributed, and adequate time was provided for completion. Out of 250 eligible students, 190 were present and consented to participate. Of these, 159 students returned completely filled questionnaires, meeting the required sample size.

Data analysis

Data were entered into Microsoft Excel and analyzed using IBM SPSS Statistics, version 21.0 (IBM Corp., Armonk, NY). Quantitative variables were expressed as mean and standard deviation (SD) for normally distributed data and as median and interquartile range (IQR) for skewed data. Categorical variables were summarized as frequencies and percentages. Associations between categorical variables were assessed using the chi-square test, and effect sizes were interpreted using Cramer’s V coefficient to determine the strength of association [12]. A p-value of <0.05 was considered statistically significant. 

## Results

The total response rate of the study was 63.6% among 250 participants. The socio-demographic characteristics of the study participants are presented in Table 1.

**Table 1:** Socio-demographic details of study participants (n = 159)

Variables	Frequency (%)
Age	
<21 years	109 (68.6%)
≥21 years	50 (31.4%)
Gender	
Male	63 (39.6 %)
Female	96 (60.4 %)
Domicile	
Urban	104 (65.4%)
Semi-urban	14 (8.8 %)
Rural	41 (25.8 %)
Board of higher secondary school	
CBSE	19 (11.9%)
Matriculation	88 (55.3%)
Samacheer	47 (29.6%)
State board	5 (3.1%)
Type of family	
Nuclear family	126 (79.2%)
Joint family	3 (1.9%)
Three-generation family	27 (17%)
Others	3 (1.9%)
Type of accommodation	
Day-scholar	43 (27%)
Hostelite	116 (73%)

Among the study participants, a significant portion of the students were under the age of 21, comprising 68.6%. Additionally, 60.4% of the students were female, and 65.4% hailed from urban areas. Furthermore, 55.3% of the students had completed their matriculation, and 79.2% belonged to nuclear families, with 73% of the students staying in the hostel. 

**Figure 1:**
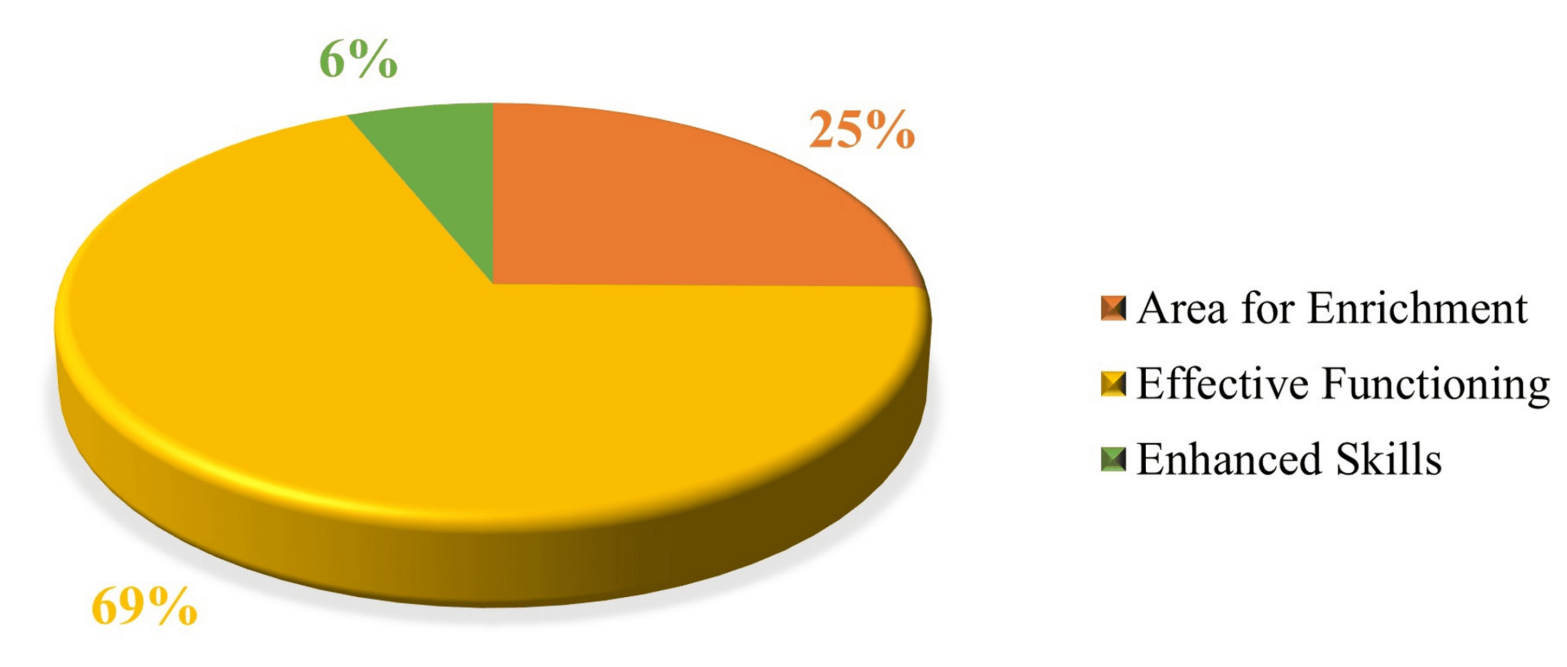
Distribution of total emotional intelligence according to its score (n = 159)

Based on the total EI scores, more than two-thirds of students (69%) had a satisfactory EI score, one-fourth (25%) had a poor EI score, and the remaining (6%) had a good EI score, indicating enhanced skills (Figure 1).

**Figure 2:**
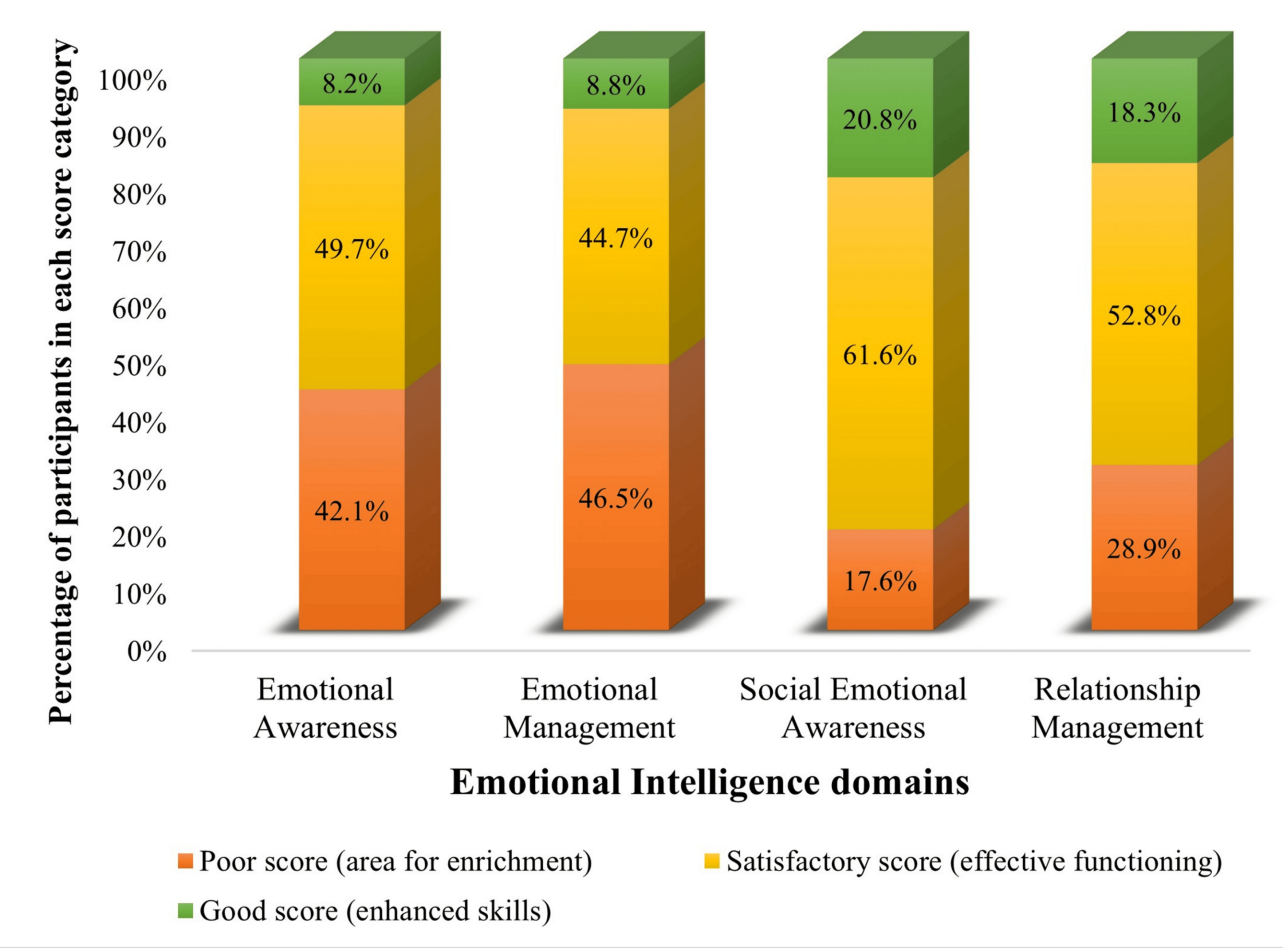
Categorization of scores across the four emotional intelligence domains (n = 159)

Across all four EI domains, the majority of individuals fall into the “satisfactory score (25-34): effective functioning” category. However, emotional awareness and emotional management exhibit a relatively higher percentage of individuals in the “poor score (0-24): area for enrichment” category compared to social emotional awareness and relationship management. Notably, social emotional awareness has the highest combined percentage of individuals demonstrating both effective functioning and enhanced skills (Figure 2).

**Figure 3:**
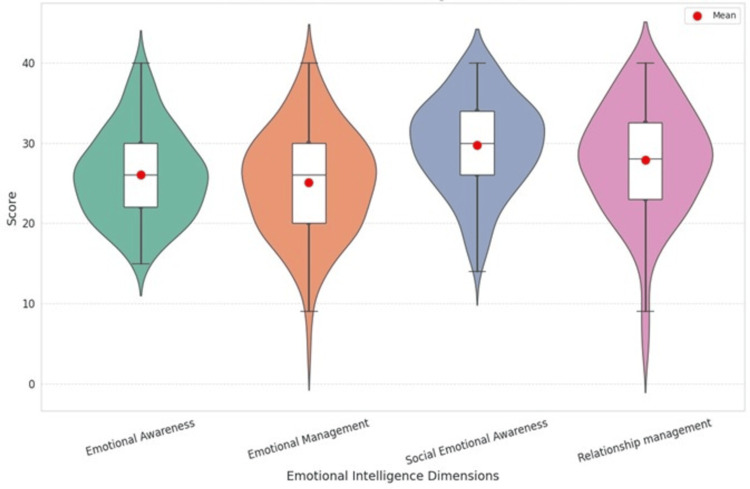
Distribution of scores across emotional intelligence domains using violin plots (n = 159)

The total EI scores ranged from 57 to 160. The normality of the data distribution was assessed using the Kolmogorov-Smirnov test, which showed that the total EI scores were normally distributed, with a mean of 108.69 and a SD of 18.35. However, the domain-wise scores showed skewed distribution, with the following median and IQR: emotional awareness = 26 (IQR: 8), emotional management = 26 (IQR: 10), social emotional awareness = 30 (IQR: 8), and relationship management = 28 (IQR: 10), as illustrated in Figure 3.

The components of feedback-seeking behavior among the study participants, namely perceived susceptibility, perceived intensity, perceived benefits, perceived barriers, and cues to action, are presented in Table 2.

**Table 2:** Feedback-seeking behavior of study participants (n = 159)

Components of feedback-seeking behavior	Agree	Neither agree nor disagree	Disagree
Perceived susceptibility (engage with feedback even if it causes discomfort)	132 (83%)	13 (8.2%)	14 (8.8%)
Perceived intensity (belief that feedback is as important for learning as lectures or tutorials)	67 (42.1%)	18 (11.3%)	74 (46.5%)
Perceived benefits (recognition of feedback as a valuable resource to support learning)	139 (87.4%)	12 (7.5%)	8 (5%)
Perceived barriers (attitude that minimal or no significant action is needed after receiving feedback)	49 (30.8%)	24 (15.1%)	86 (54.1%)
Cues to action (perception of feedback as motivating)	132 (83%)	19 (11.9%)	8 (5%)

**Figure 4:**
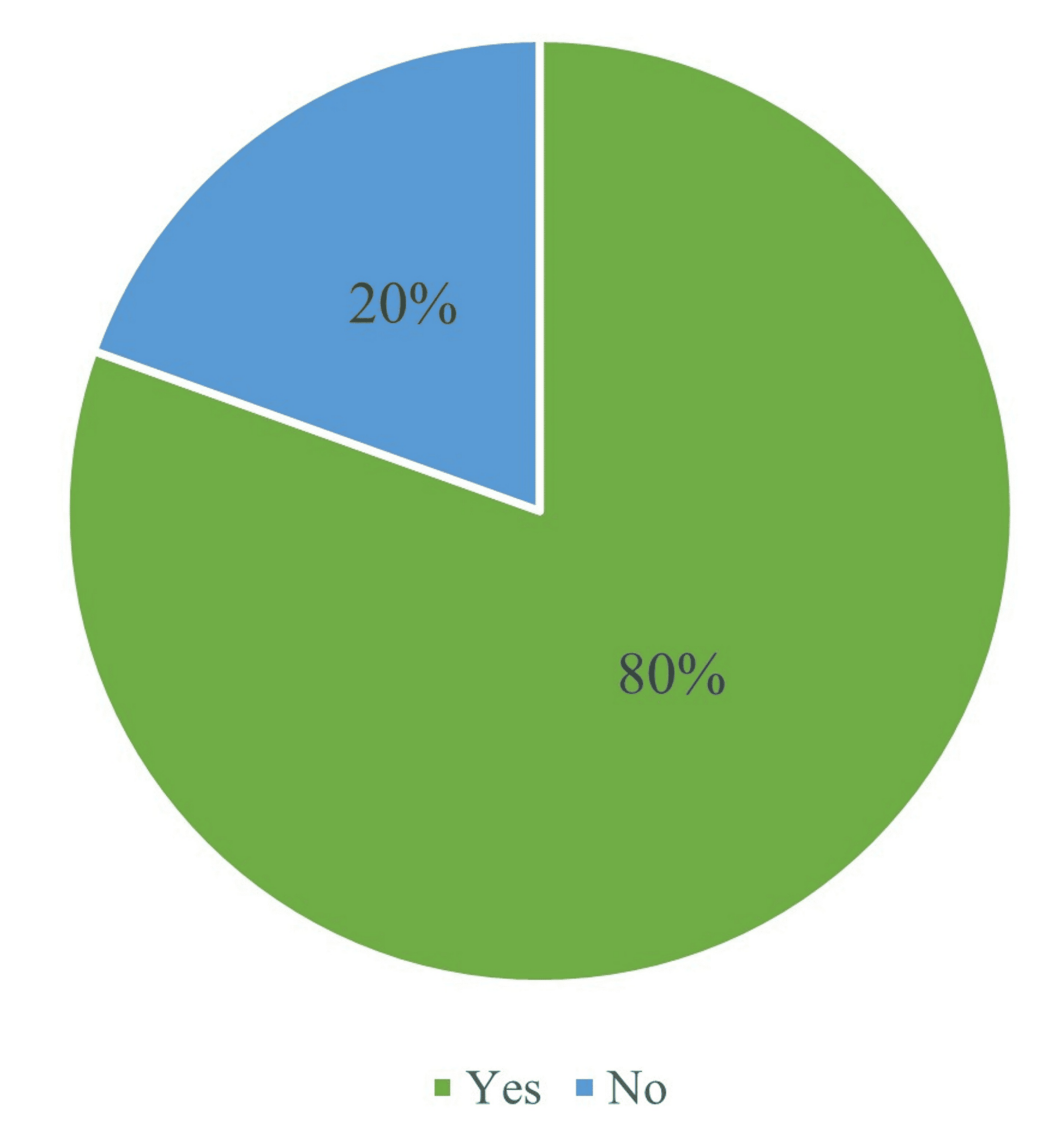
Self-efficacy (feedback-seeking intent) (n = 159) Yes: always, often, and sometimes; No: rarely and never

The self-efficacy component of feedback-seeking behavior is illustrated in Figure 4.

A moderate and statistically significant association was observed between gender and relationship management, with females (56.3%) scoring higher than males (χ² value = 6.462; Cramer’s V = 0.202; p = 0.040). Similarly, a moderate and statistically significant association was found between the type of accommodation and emotional management, with day-scholars (55.8%) scoring better than hostelites (χ² value = 6.904; Cramer’s V = 0.208; p = 0.032), as shown in Table 3.

**Table 3:** Association between emotional intelligence domains and socio-demographic factors using Cramer’s V coefficients (n = 159) Cramer’s V: 0.20-0.40 indicates a moderate association. *Values denote statistical significance at p < 0.05. ^#^Categories: age (<21 years and ≥21 years), gender (male and female), domicile (urban, semi-urban, and rural), board of higher secondary schooling (CBSE, matriculation, samacheer, and state board), type of family (nuclear family, joint family, three-generation family, and others), and type of accommodation (day-scholar and hostelite).

Socio-demographic factors (categories)^#^	Emotional intelligence domains			
	Emotional awareness	Emotional management	Social emotional awareness	Relationship management
Age	0.11	0.13	0.083	0.178
Gender	0.19	0.076	0.137	0.202*
Domicile	0.093	0.13	0.139	0.128
Board of higher secondary school	0.102	0.111	0.117	0.126
Type of family	0.155	0.112	0.111	0.122
Type of accommodation	0.037	0.208*	0.102	0.086

Feedback was found to be motivating for students under the age of 21 (85.3%) and female students (88.5%), with a moderately significant association (χ² value = 7.493; Cramer’s V = 0.217; p = 0.024 and χ² value = 6.419; Cramer’s V = 0.201; p = 0.040, respectively). Students from nuclear families demonstrated a strong desire to engage with feedback, even if it made them feel anxious (79.5%; χ² value = 16.022; Cramer’s V = 0.224; p = 0.014). They also considered that feedback is as crucial as lectures or tutorials for their learning, with a moderately significant association (80.6%; χ² value = 14.696; Cramer’s V = 0.215; p = 0.023), as shown in Table 4.

**Table 4:** Association between feedback-seeking behavior and socio-demographic factors using Cramer’s V coefficients (n = 159) Cramer’s V: 0.20-0.40 indicates a moderate association. *Values denote statistical significance at p < 0.05. ^#^Categories: age (<21 years and ≥21 years), gender (male and female), domicile (urban, semi-urban, and rural), board of higher secondary schooling (CBSE, matriculation, samacheer, and state board), type of family (nuclear family, joint family, three-generation family, and others), and type of accommodation (day-scholar and hostelite).

Socio-demographic factors (categories)^#^	Components of feedback-seeking behavior					
	Engage with feedback even if it made you feel worried	Important for learning as lectures or tutorials	Resource to support learning	Does not take much action after receiving it	Feedback is motivating	Feedback-seeking intent
Age	0.132	0.169	0.105	0.084	0.217*	0.026
Gender	0.134	0.055	0.051	0.102	0.201*	0.15
Domicile	0.12	0.107	0.096	0.093	0.097	0.077
Board of higher secondary school	0.133	0.166	0.111	0.163	0.094	0.194
Type of family	0.224*	0.215*	0.058	0.1	0.071	0.186
Type of accommodation	0.028	0.134	0.055	0.127	0.139	0.106

Students with better social emotional awareness (91.8%) and effective relationship management (94%) agreed that feedback was a key support for learning, with moderately significant associations (χ² value = 18.481; Cramer’s V = 0.241; p = 0.002 and χ² value = 14.577; Cramer’s V = 0.214; p = 0.006, respectively). Students with effective relationship management (83.3%) considered feedback to be motivating, and this showed a weak but statistically significant association (χ² value = 11.342; Cramer’s V = 0.189; p = 0.023). Furthermore, those with enhanced relationship management skills (62.1%) reported higher feedback-seeking intention, with a moderately significant association (χ² value = 10.697; Cramer’s V = 0.259; p = 0.005), as presented in Table 5.

**Table 5:** Association between emotional intelligence and feedback-seeking behavior using Cramer’s V coefficients (n = 159) Cramer’s V: 0.10-0.20 indicates a weak association, while 0.20-0.40 indicates a moderate association. *Values denote statistical significance at p < 0.05.

Emotional intelligence domains	Components of feedback-seeking behavior					
	Engage with feedback even if it made you feel worried	Important for learning as lectures or tutorials	Resource to support learning	Does not take much action after receiving it	Feedback is motivating	Feedback-seeking intent
Emotional awareness	0.071	0.143	0.12	0.165	0.095	0.115
Emotional management	0.037	0.163	0.066	0.156	0.067	0.114
Social emotional awareness	0.099	0.09	0.241*	0.135	0.171	0.105
Relationship management	0.121	0.105	0.214*	0.111	0.189*	0.259*

## Discussion

Over two-thirds (69%) of the study participants demonstrated a satisfactory EI score, which is higher than that reported in other studies. For instance, George et al.’s study conducted among medical students in Central Kerala found that 63.3% had satisfactory EI scores [13], while a study among postgraduate medical students in Central India reported only 58% [4]. The relatively higher EI scores among undergraduates in our study, compared to the findings of Ukey et al. among postgraduates, may suggest that EI remains relatively stable or even higher during undergraduate training, possibly due to a structured learning environment, institutional emphasis on communication and soft skills, and peer support [4]. Furthermore, this provides a strong rationale for instituting efforts to promote a feedback-seeking culture as early as possible during the undergraduate period, when EI levels are relatively higher.

The total EI scores in our study ranged from 57 to 160, with a mean of 108.69 (SD = 18.35). This aligns closely with Sundararajan and Gopichandran’s study in Chennai (mean = 107.58; SD = 16.44) [1] and with another study among medical postgraduates in Central India (mean = 107.02; SD = 25.08) [4].

Our findings also affirm that females demonstrated significantly better relationship management than males, consistent with previous evidence by George et al. and Sánchez-Núñez et al., which suggests superior interpersonal and empathetic skills among women [13,14]. This supports observations by Sundararajan and Gopichandran and Venkatappa et al., where females showed higher EI and better responses to emotional scenarios, potentially leading to enhanced clinical care delivery [1,2]. In contrast, male postgraduate students in Central India displayed better emotional awareness and management, possibly reflecting gender-based differences in coping strategies influenced by societal norms [4]. However, Vasefi et al., in Iran, found no significant gender differences in EI [3]. These variations across studies highlight the influence of biological, geographical, cultural, and societal factors in shaping gender-related differences in emotional competencies.

Day-scholars in our study exhibited significantly better emotional management skills compared to hostelites, a finding consistent with studies by Waddar et al. among postgraduate students and by Sunny et al. in Kerala [15,16]. This trend could be attributed to the fact that day-scholars benefit from stronger familial bonds, positive role models, and fostering better emotional regulation. In contrast, Sinha et al.’s study reported that hostelites face unique challenges such as adjusting to diverse peer groups, managing independence, and coping with academic and social demands in an unfamiliar environment [17]. These findings underscore the importance of environmental and familial factors in shaping EI and highlight the need for targeted interventions, such as mentorship programs, peer counseling, and stress management workshops, especially within hostel premises, to bridge the gap in emotional competencies among hostel-based students.

In our study, students under 21 years of age and females found feedback to be significantly motivating, consistent with the findings of Jones et al., where women sought feedback more often for motivational purposes [18]. Students from nuclear families showed a strong inclination to engage with feedback, even when it induced anxiety, and valued it as essential as lectures or tutorials. These findings highlight the importance of considering demographic factors such as age, gender, and family dynamics when designing effective feedback strategies. Tailoring feedback to these factors could enhance student engagement and improve learning outcomes. This calls for the incorporation of affect-aware feedback strategies, rather than relying solely on cognitive feedback, to address the diverse challenges faced by students from varied demographic backgrounds [19,20].

Students with higher social-emotional awareness and relationship management skills significantly viewed feedback as a key support for learning. This highlights that students who are emotionally aware and skilled in managing relationships better appreciate the value of feedback and utilize it to enhance their learning. Additionally, students with strong relationship management skills were more likely to seek feedback and found it more motivating, indicating that effective interpersonal skills drive proactive feedback-seeking behavior. Overall, these results emphasize the critical role of EI and relationship management in fostering a positive feedback culture. This is consistent with Yang et al.’s study, which underscores a strong association between EI and feedback-seeking behavior [9]. Students with higher feedback self-efficacy demonstrate greater confidence in acting on feedback, while those with enhanced social awareness are more inclined to maintain positive relationships with teachers, thereby encouraging continued engagement with feedback. Furthermore, students often seek clarification from teachers or emotional support from peers to help process feedback both cognitively and emotionally [9].

Pinasthika and Findyartini’s findings among medical students revealed that motivation, self-assessment, learning styles, and awareness of their strengths and weaknesses were key factors influencing feedback-seeking behavior [5]. This underlines the importance of motivation and self-awareness in fostering a feedback-driven approach for continuous academic improvement. Such proactive feedback-seeking behavior can lead to higher achievement and enhanced learning outcomes, highlighting that students who embrace feedback as a tool for growth tend to perform better.

Strengths and limitations

The validity of the findings was assured by conducting the survey anonymously and using a standardized questionnaire to assess EI. Participation was voluntary, with no repercussions for declining to participate or for providing critical responses. It was succinctly stated that the purpose of the survey was solely for educational improvement. However, the findings of our study may not be generalizable, as the sample was confined to third-year undergraduate medical students from a single medical college. Furthermore, being a cross-sectional study, causal associations between EI and feedback-seeking behavior could not be established. Socio-demographic variables alone cannot fully explain the determining factors, and unmeasured confounding variables, such as academic stress, prior feedback experiences, and other psychosocial factors, may have influenced the findings. Furthermore, the reliance on self-reported responses may introduce the possibility of subjective bias. This study also did not explore the qualitative aspects of EI and feedback-seeking behavior among medical students.

## Conclusions

Most students achieved a satisfactory EI score, indicating effective functioning. However, one-fourth of the students had poor EI scores, highlighting a potential area for enrichment that requires attention and development. Relationship management was significantly better in females compared to males, and day-scholars demonstrated significantly better emotional management skills than hostelites. Demographic factors such as age, gender, and family structure were significantly associated with feedback-seeking behavior. Students who viewed feedback as a key support for learning showed significantly higher social-emotional awareness and relationship management. Feedback was considered highly motivating to students who were better at managing their relationships, and the intention to seek feedback was significantly higher among those with stronger relationship management skills. Thus, enhancing EI is a crucial component in fostering a culture of feedback-seeking among medical students. 

Recommendations

This study emphasizes the need to integrate EI training into undergraduate medical education. The attitude, ethics, and communication (AETCOM) module serves as a platform to impart education on communication skills, empathy, EI, and ethics. Incorporating cognitive reflection exercises, practical role-play scenarios, and conflict resolution methods can effectively train medical students in these areas. Enhancing students’ EI can improve their feedback-seeking behavior and contribute to the development of more empathetic and effective physicians in the future.

## Appendices

Study questionnaire

(A) Socio-demographic details:

Name:

Age:

Gender: Male/Female 

Domicile: Urban/semi-urban/rural

Board of higher secondary school: CBSE/Matriculation/Samacheer/State board

Type of family: Nuclear/Joint/three generation/others

Type of accommodation: Day-scholar/hostelite

(B) The Quick Emotional Intelligence Self-Assessment:

Rank each statement as follows: 0 (never), 1 (rarely), 2 (sometimes), 3 (often), 4 (always)

Emotional awareness:

1. My feelings are clear to me at any given moment (0/1/2/3/4)

2. Emotions play an important part in my life (0/1/2/3/4)

3. My moods impact the people around me (0/1/2/3/4)

4. I find it easy to put words to my feelings (0/1/2/3/4)

5. My moods are easily affected by external events (0/1/2/3/4)

6. I can easily sense when I’m going to be angry (0/1/2/3/4)

7. I readily tell others my true feelings (0/1/2/3/4)

8. I find it easy to describe my feelings (0/1/2/3/4)

9. Even when I’m upset, I’m aware of what’s happening to me (0/1/2/3/4)

10. I am able to stand apart from my thoughts and feelings and examine them (0/1/2/3/4)

Emotional management:

1. I accept responsibility for my reactions (0/1/2/3/4)

2. I find it easy to make goals and stick with them (0/1/2/3/4)

3. I am an emotionally balanced person (0/1/2/3/4)

4. I am a very patient person (0/1/2/3/4)

5. I can accept critical comments from others without becoming angry (0/1/2/3/4)

6. I maintain my composure, even during stressful times (0/1/2/3/4)

7. If an issue does not affect me directly, I don’t let it bother me (0/1/2/3/4)

8. I can restrain myself when I feel anger towards someone (0/1/2/3/4)

9. I control urges to overindulge in things that could damage my well-being (0/1/2/3/4)

10. I direct my energy into creative work or hobbies (0/1/2/3/4)

Social emotional awareness:

1. I consider the impact of my decisions on other people (0/1/2/3/4)

2. I can tell easily tell if the people around me are becoming annoyed (0/1/2/3/4)

3. I sense it when a person’s mood changes (0/1/2/3/4)

4. I am able to be supportive when giving bad news to others (0/1/2/3/4)

5. I am generally able to understand the way other people feel (0/1/2/3/4)

6. My friends can tell me intimate things about themselves (0/1/2/3/4)

7. It genuinely bothers me to see other people suffer (0/1/2/3/4)

8. I usually know when to speak and when to be silent (0/1/2/3/4)

9. I care what happens to other people (0/1/2/3/4)

10. I understand when people’s plans change (0/1/2/3/4)

Relationship management:

1. I am able to show affection (0/1/2/3/4)

2. My relationships are safe places for me (0/1/2/3/4)

3. I find it easy to share my deep feelings with others (0/1/2/3/4)

4. I am good at motivating others (0/1/2/3/4)

5. I am a fairly cheerful person (0/1/2/3/4)

6. It is easy for me to make friends (0/1/2/3/4)

7. People tell me I am sociable and fun (0/1/2/3/4)

8. I like helping people (0/1/2/3/4)

9. Others can depend on me (0/1/2/3/4)

10. I am able to talk to someone down if they are very upset (0/1/2/3/4)

(C) Feedback-seeking behavior:

1. I want to engage with my feedback even if it makes me feel worried to read it (agree/neither agree nor disagree/disagree)

2. Feedback is as important as lectures or tutorials (agree/neither agree nor disagree/disagree)

3. Feedback is a resource to support learning (agree/neither agree nor disagree/disagree)

4. You should not take much action when you receive feedback (agree/neither agree nor disagree/disagree)

5. Feedback is motivating (agree/neither agree nor disagree/disagree)

6. How often do you have the intention to seek feedback from someone? (always/often/sometimes/rarely/never)

Signature of the participant
